# Development and validation of a structured survey questionnaire on knowledge, attitude, preventive practice, and treatment-seeking behaviour regarding dengue among the resident population of Sabah, Malaysia: an exploratory factor analysis

**DOI:** 10.1186/s12879-021-06606-6

**Published:** 2021-08-31

**Authors:** Rhanye Mac Guad, Ernest Mangantig, Wah Yun Low, Andrew W. Taylor-Robinson, Meram Azzani, Shamala Devi Sekaran, Maw Shin Sim, Nornazirah Azizan

**Affiliations:** 1 Department of Pharmaceutical Life Sciences, Faculty of Pharmacy, Universiti Malaya, 50603 Kuala Lumpur, Malaysia; 2 Department of Biomedical Science and Therapeutics, Faculty of Medicine and Health Sciences, Universiti Malaysia Sabah, 88400 Kota Kinabalu, Malaysia; 3 Regenerative Medicine Cluster, Advanced Medical and Dental Institute, Universiti Sains Malaysia, Bertam, 13200 Kepala Batas, Pulau Pinang Malaysia; 4 Faculty of Medicine, Universiti Malaya, 50603 Kuala Lumpur, Malaysia; 5 Asia-Europe Institute, Universiti Malaya, 50603 Kuala Lumpur, Malaysia; 6 Infectious Diseases Research Group, School of Health, Medical and Applied Sciences, Central Queensland University, Brisbane, QLD 4000 Australia; 7 College of Health and Human Sciences, Charles Darwin University, Casuarina, NT 0810 Australia; 8 College of Health Sciences, Vin University, Gia Lam District, Hanoi, Vietnam; 9 Department of Community Medicine, School of Medicine, Faculty of Medicine, Bioscience and Nursing, MAHSA University, 42610 Selangor, Malaysia; 10 Faculty of Medicine and Health Sciences, UCSI University, 56000 Kuala Lumpur, Malaysia; 11 Department of Pathobiology and Medical Diagnostic, Faculty of Medicine and Health Science, Universiti Malaysia Sabah, 88400 Kota Kinabalu, Malaysia

**Keywords:** Knowledge, Attitude, Practice, Dengue, Survey, Validation, Sabah Malaysia

## Abstract

**Background:**

Several studies have reported a significant association of knowledge, attitude and preventive practice (KAP) regarding dengue infection among community’s resident in endemic areas. In this study we aimed to assess and develop a reliable and valid KAP survey on the subject of dengue that is suitable for the resident population of Sabah, Malaysia.

**Methods:**

A community-based cross-sectional study was conducted from October 2019 to February 2020 involving 468 respondents. Information on the socio-demographic characteristics of the participants (six items), their KAP (44, 15 and 18 items on knowledge, attitude and practice, respectively) and treatment-seeking behaviour (five items) towards dengue was collected using a structured questionnaire. Data analysis was performed using SPSS and R software in the R Studio environment. The knowledge section was analysed by two-parameter logistic item response theory (2-PL IRT) using ltm package. The construct validity and reliability of items for sections on attitude, practice and treatment-seeking behaviour were analysed using psy package.

**Results:**

For the knowledge section, only 70.5% (31/44) of items were within or close to the parameter acceptable range of −3 to + 3 of difficulty. In terms of discrimination, 65.9% (29/44) of items were within or close to the acceptable range of 0.35 to 2.5, and 24 items (54.5%) failed to fit the 2-PL IRT model (P < 0.05) after assessing by goodness-of-fit analysis. Only eight items were reliable and retained in the attitude section with a Kaiser–Meyer–Olkin (KMO) test value of > 0.7, while based on the communalities, 11 items in the attitude section were excluded due to very low h2, factor loading values and low correlation with the total (< 0.5). The practice section was found suitable for factor analysis because the KMO value was > 0.7. The communalities of the practice section showed that seven items had low h2 values (< 0.3), which were therefore excluded from further analysis, and only 11 items were retained.

**Conclusions:**

The KAP items retained in the final version of the survey were reliable and valid to be use as a questionnaire reference when conducting future similar studies among the population of Sabah.

**Supplementary Information:**

The online version contains supplementary material available at 10.1186/s12879-021-06606-6.

## Background

Dengue, a mosquito-borne viral disease of humans, is becoming a major global public health concern and socioeconomic burden, especially in tropical and subtropical countries because of the increasingly high incidence of infection [1]. This includes the Southeast Asian nation of Malaysia that occupies parts of the Malay Peninsula and the island of Borneo. In this country of 32 million people there has been a recent steep rise in the number of dengue cases reported annually, from 46,171 in 2010 to 83,849 in 2017 [2, 3]. The largest ever outbreak was reported in 2014, with 108,698 confirmed cases, which equated to an incidence rate of 361 per 100,000, and 215 deaths [4, 5]. The incidence of dengue varies between the 13 states and three federal territories that comprise Malaysia due to several associated risk factors. According to current national statistics on dengue, Sabah, located in East Malaysia on the northern portion of Borneo, recorded a cumulative 3615 dengue cases between December 2019 and June 2020, making it the state with the fourth highest incidence, ranking only behind the more developed states of Kuala Lumpur, Selangor and Johor [4]. Patterns of dengue transmission in Sabah are associated with a rapid rate of urbanization in close proximity to disturbed forest environments, thereby providing a risk of spill-over of sylvatic pathogens to human populations [6].

In order to combat the escalation of dengue in Malaysia considerable measures have been undertaken to prevent or limit the risk factors of infection among the population. The most powerful prevention strategy is through health education and community participation. It is crucial to assess local communities on their knowledge, attitude and practice (KAP) towards dengue prevention practices so as to achieve an effective health intervention. While the current investigation is the first of its kind to be performed in East Malaysia several questionnaire-based studies conducted recently in Peninsular Malaysia (separated from Borneo by the South China Sea) have indicated a significant association between KAP and dengue infection. For instance, persons who possess a high level of knowledge of dengue demonstrate a significantly better attitude and practice towards prevention measures [7]. There is also contradictory evidence to indicate that having knowledge of dengue is not certain to make an individual adopt recommended preventive behaviours [8]. Such a discrepancy may be explained by several factors including a person’s lack of exposure to a dengue awareness program [9], their misconception due to inadequate knowledge [10] or traditional beliefs [11], as well as unreliable/improper methodology of data collection or questionnaire use [12].

It has been shown that measurement properties are population-specific and vary depending upon the setting and context of the questionnaire used [13]. An existing questionnaire prepared for one population may not be applicable to another in a different country or region with a distinct set of socio-cultural, health system, economic and political contexts. For instance, Sabah located in east Malaysia has a diverse multiethnicity of more than 40 ethnic groups, within which there are over 200 sub-ethnic groups each having its own language, culture and belief system. Additionally, according to the most recent national census (2015), Sabah has the highest non-Malaysian population (25.6%) [14]. Interestingly, the hotspots for highest prevalence of dengue in the Sabah area are occupied predominantly by the non-Malaysian population. This unique combination of Malaysian and non-Malaysian ethnic groups makes Sabah an advantageous site for dengue investigation. There is no gold standard questionnaire for the assessment of KAP due to the heterogeneity that exists in each population. Thus, the development and validation of a KAP questionnaire that is appropriate for the Sabah population would be extremely beneficial. However, a tried and tested survey validation process for Malaysian populations is currently lacking, particularly for use among the Sabah population, and so this also requires evaluation and documenting.

The majority of surveys that are conducted by questionnaire are reported either as a piloted, pre-tested questionnaire or a Cronbach’s alpha single-administration test score reliability coefficient as a measure of internal consistency. Thus, a rigorous methodology is required to examine the degree an instrument is affected by measurement error (reliability) and by the construct it intends to measure (validity) [15]. The types of validity include concurrent validity (measuring the degree that it purports to measure) and criterion validity (measuring the degree to which the results of the questionnaire are an adequate reflection of a gold standard). Hence, with the aim to assist in planning an effective health intervention towards dengue infection, an accurate measurement of psychometric properties of the KAP questionnaire that is specific to the Sabah population is needed. The present survey describes the procedures involved in the development and assessment of the reliability and validity of this KAP questionnaire.

## Methods

### Ethics approval

The survey study was approved by the Universiti Malaysia Sabah ethics committee [Ethics reference number: (53) dlm.JKN (SB)100-13] and complied with the Declaration of Helsinki. Permission to interview the respondents was obtained from the district police station [KPB Kota Kinabalu 10/8/11]. All respondents included in this study gave written informed consent before participation. Those individuals who participated remotely by completing an online survey form (Google) provided informed consent form immediately prior to clicking the “next page” button to start the survey.

### Study design and sample size

The survey study was designed as a cross-sectional measurement to develop and validate a questionnaire on KAP and treatment-seeking behaviour regarding dengue among the Sabah resident population. It was divided into a pilot study and a validation study involving 30 and 468 respondents, respectively. The respondent inclusion criteria included being 18 years of age or older, the ability to read Sabah Malay creole and not being a health professional. The sample size required for verification of the survey was based on the ratio of the number of items in a questionnaire to the number of respondents, with 1:10 as a preferred rule-of-thumb [16–18]. A sample size of 380 was required for exploratory factor analysis (EFA) whenever ten or more items were expected to have a factor loading of 0.4 [19]. The sample size was inflated to 494 in order to account for an anticipated 30% non-response rate. The required sample size for two-parameter logistic item response theory (2-PL IRT) followed that for EFA because there is no definitive size for IRT, although it may range from 100 to 500 samples [20].

### Development of questionnaire items

A thorough literature review was conducted to collate and review existing questionnaires related to KAP and treatment-seeking behaviour on dengue. An assessment of the level of difficulty and adequacy of the questionnaire was performed by a panel of experts comprising an epidemiologist, infectious disease specialists, a statistician, and a biomedical scientist. From this process, an initial selection of 110 items was reduced to a total of 97. The items chosen for the questionnaire were adjusted according to the local setting and divided into four primary sections, as shown in Table 1. These were determined as follows: (1) Demographic factors (six items) examining demographic and socio-economic characteristics; (2) Knowledge (44 items), defined as respondents’ knowledge of dengue prevention, including possible causes of dengue recurrence, symptoms, transmission, treatment and prevention, requirement for dengue precautions and recommended/non-recommended practices. This section consisted of true/false response options with a “don’t know” category provided for each question. A correct answer (such as ‘mosquito’ as the transmission vector of dengue) received one point whereas an incorrect answer (such as ‘Aedes aegypti habitually bites at midday’) and a “don’t know” response each received zero points; (3) Attitude (15 items), defined as respondents’ opinions about dengue prevention, awareness, daily care and socio-cultural perspective. This section consisted of agree/disagree response options with a “not sure” category provided for each question. Responses deemed to be either good or acceptable were awarded one point each, while all other responses received zero points; (4) Practice (18 items), defined as respondents’ practice towards dengue prevention, such as action taken to avoid occurrence of infection. This section consisted of yes/ no response options with “not sure” and “not related” categories provided for each question. A point score for each respondent was determined based on their good practice, with none awarded for poor practice. A “do not have” answer did not count towards the cumulative score and the total score (denominator) was commensurately reduced. A secondary section of the questionnaire assessed treatment-seeking behaviour of respondents and consisted of a final five items. The questions used are included in an Additional file 1.

**Table 1:** Details of questionnaire sections on sociodemographic, KAP and treatment-seeking behaviour regarding dengue

Section	No. of items	Concepts measured	Response options
Sociodemographic	6	Age, gender, race, marital status, level of education, occupation	Closed-ended, multiple-choice
Knowledge	44	General knowledge of dengue: aspects related to symptoms and signs, causes, exposure, and treatment	True/false/don’t know
			1 = Correct answer
			0 = Incorrect/don’t know
Attitude	15	Attitudes towards dengue prevention	1 = Strongly agree
			2 = Agree
			3 = Not sure
			4 = Disagree
			5 = Strongly disagree
Preventive practice	18	Practices to reduce *Aedes* mosquitoes and larvae	1 = Yes
			2 = No
			3 = Not sure
			4 = Not related
Treatment-seeking behaviour	5	Behaviour towards seeking treatment for dengue	1 = Yes
			2 = No

### Pilot study

After assessment of the content validity by the expert panel, the pilot questionnaire was pre-tested on 30 respondents recruited randomly based on local acquaintances of the researchers (conducted in September 2019). The selection criteria included Sabahan (residents of Sabah) aged 18 years old and above, no known history of dengue infection, literate in Sabah Malay creole and working in non-medical-related professions. Respondents were recruited either physically or remotely. The former individuals were required to complete the questionnaire by hand (self-administered) on a hard copy, while the latter individuals did so online by using a Google survey form. For the self-administration method, a trained research assistant was assigned to check all items on-site before ensuring the return of each completed questionnaire to the researchers. The response rate was 96.67% (29/30 participants). Respondents took an average of 15 min to complete the questionnaire.

### Validation study

**Fig. 1:**
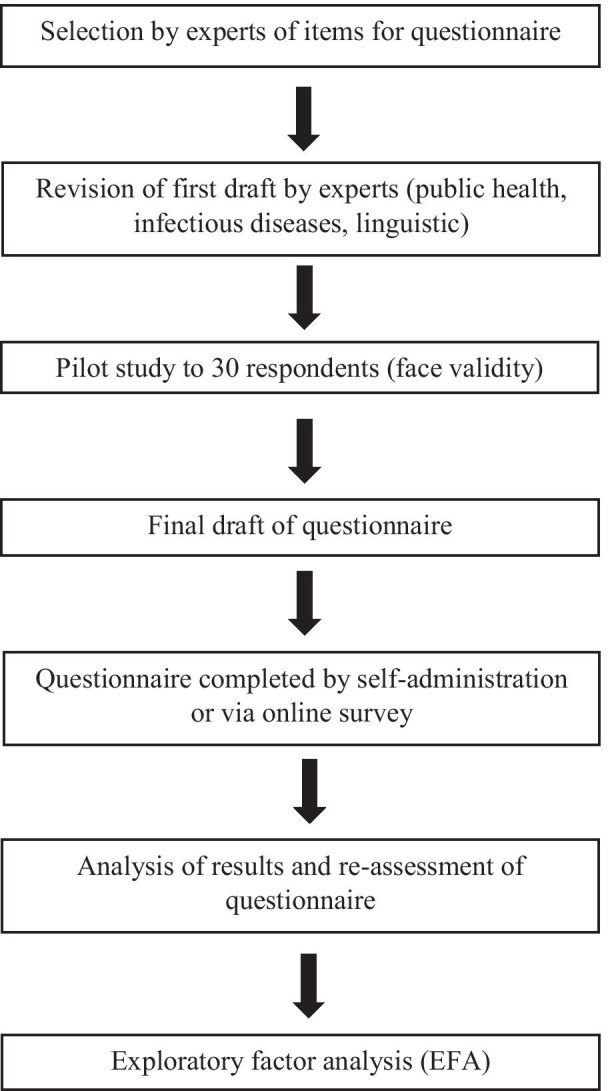
Flowchart of questionnaire development and validation

Based on the findings of the pilot study, a full validation study using IRT and EFA was conducted from October 2019 to February 2020. Respondents were recruited as described for the pilot study, either by convenience sampling, undertaken in Kota Kinabalu, the state capital of Sabah (self-administration), or through an online Google survey. Remote respondents came from all five divisions of Sabah state: Interior, Kudat, Sandakan, Tawau and West Coast. The timeline of questionnaire development and validation is shown in Fig. 1.

### Statistical analysis

Data analysis was performed using SPSS Statistics for Windows (version 24.0) (IBM Corp., released 2011) and R software (version 3.3.2) in the R Studio environment. As the knowledge section of the questionnaire consisted of unidimensional items with dichotomous responses (correct/incorrect), it was analysed by 2-PL IRT using ltm package version 1.0 [21]. The metrics used to assess knowledge items were difficulty index (proportion of respondents who answered the item correctly) and discrimination index (how the ‘overall best’ respondents compared to the ‘overall worst’ respondents with regard to a particular item). A difficulty index in the range of − 3 to 3 and a discrimination index in the range of 0.35 to 2.5 were considered acceptable [22]. The Chi-square goodness-of-fit test determined if the observed frequencies fitted a specified distribution for each item [23]. IRT analysis makes a strong assumption of unidimensionality, a property that is used to determine whether items are suitable to be summed as a total score. Modified parallel analysis determined unidimensionality, with P-value ≥ 0.05 indicating that the assumption was met [24].

Attitude, practice and treatment-seeking behaviour were analysed by EFA using psy package version 1.8.4 to assess the construct validity and reliability of items in each section [25]. These were treated as continuous responses to allow evaluation of the dimensionality (number of factors) of items. The Kaiser-Meyer-Olkin (KMO) test measured the relative degree of correlation for a set of items in the questionnaire. A KMO value of > 0.7 indicated the adequacy and suitability of data for factor analysis. Communality (h2) is the proportion of variance that is shared among a set of items, for which an h2 value > 0.3 was considered acceptable. The principal axis factoring extraction method with oblimin rotation was applied in the EFA. Eigenvalues > 1.0 and a scree plot inspection determined the number of factors extracted. Factor loadings of > 0.4 were considered acceptable [26]. For indicators of internal consistency, a corrected item-total correlation of > 0.5 and a Cronbach’s alpha score > 0.7 were considered reliable [27].

## Results

### Sociodemographic details of respondents

The demographic characteristics of the 468 respondents who participated in this study are shown in Table 1. The most common age range of participants was 18–30 years old (41.2%) with a mean age of 28.43 (± 9.54) years. Interestingly, most were Kadazan-Dusun (62.3%), an ethnic group indigenous to Sabah, and predominantly females (60.5%). A high proportion of respondents received tertiary education (68.6%), while 26.5% completed secondary education.

### Knowledge

Of a total of 44 items several were considered problematic after analysing by IRT (Table 2). Pertaining to the difficulty parameter only 31 items were within or close to the acceptable range of −3 to + 3. Two items (K1.9 and K6.8) each exceeded the cut-off value of + 3 by a large margin, indicating that the items were too difficult. Additionally, one item (K2) was, by 2.5, slightly above the cut-off margin. Eleven items (K1.1, K1.4, K1.12, K1.18, K3.3, K4.2, K6.1, K6.2, K6.4, K6.5 and K11) had a difficulty index below the -3 cut-off value, indicating that these items were relatively easy and should be considered for exclusion. In terms of discrimination, 29 items were within or close to the acceptable range of 0.35 to 2.5. In contrast, eight items (K1.2, K1.9, K1.10, K1.11, K1.15, K1.16, K1.17 and K6.3) and two items (K2 and K6.8) had a lower discrimination index by a small and large margin, respectively. Only three items (K4.1, K4.3 and K4.4) were higher than the cut-off value of 2.5 by a large margin.

**Table 2:** Sociodemographic characteristics of respondents

Sociodemographic characteristic	n (%)
Age (years)	
18–30	190 (41.2)
31–40	120 (26.0)
41–50	95 (20.6)
51–60	44 (9.5)
61–70	11 (2.4)
Sex	
Female	283 (60.5)
Ethnicity	
Kadazan-Dusun	292 (62.3)
Bajau	50 (10.7)
Rungus	25 (5.3)
Sabah Malay	19 (4.1)
Brunei	13 (2.8)
Bugis	13 (2.8)
Other	56 (12.0)
Marital status	
Married	273 (58.3)
Single	190 (40.6)
Other	5 (1.2)
Level of education	
College/university	321 (68.6)
Secondary school	124 (26.5)
Primary school	17 (3.6)
No schooling	6 (1.3)
Occupation	
Employed	257 (54.9)
Self-employed	74 (15.8)
Not working	112 (23.7)
Student	14 (2.8)

The item goodness-of-fit statistics showed that 24 items did not fit well the 2-PL IRT model (P-value < 0.05). Furthermore, all items did not support the unidimensionality assumption of the model, as evidenced by modified parallel analysis (P-value = 0.01; Table 3). Based on these findings, in order to identify the best items representing knowledge that supports the unidimensionality assumption (P-value ≥ 0.05) the IRT analysis was rerun with several models using a different combination of items. Model 6 was selected in consideration of the importance of several poor fit items but with acceptable difficulty and discrimination values (Table 4). Hence, the final number of items in the knowledge section was 25, with an average Cronbach’s alpha score of 0.75 (95% CI 0.72–0.78) (Table 5).

**Table 3:** Results of IRT analysis of the knowledge section

Item	Percentage correct response	Difficulty index	Discrimination index	Chi-square value	*P*
K1.1	0.96	−5.24	0.66	10.34	0.242
K1.2	0.86	−7.58	0.24	15.34	0.053
K1.3	0.74	−3.15	0.36	10.59	0.226
K1.4	0.85	−4.65	0.39	18.65	**0.017**
K1.5	0.81	−2.79	0.59	31.00	** < 0.001**
K1.6	0.69	−2.37	0.36	25.69	**0.001**
K1.7	0.43	0.70	0.35	35.38	** < 0.001**
K1.8	0.40	0.82	0.45	47.58	** < 0.001**
K1.9	0.21	10.1	0.13	38.56	** < 0.001**
K1.10	0.54	−1.20	0.15	16.91	**0.031**
K1.11	0.61	−2.48	0.20	17.69	**0.024**
K1.12	0.31	1.31	0.61	10.36	0.241
K1.13	0.22	1.81	0.73	5.99	0.648
K1.14	0.29	1.95	0.45	23.00	**0.003**
K1.15	0.49	0.01	0.21	17.71	**0.023**
K1.16	0.36	3.07	0.18	18.96	**0.015**
K1.17	0.38	2.27	0.20	16.01	**0.042**
K2	0.47	5.52	0.02	2.76	0.948
K3.1	0.56	−0.27	2.62	27.21	**0.001**
K3.2	0.58	−0.34	2.27	28.72	** < 0.001**
K3.3	0.98	−7.33	0.54	6.36	0.607
K4.1	0.31	0.36	7.69	9.34	0.314
K4.2	0.92	−5.86	0.44	13.23	0.104
K4.3	0.30	0.37	9.37	9.19	0.327
K4.4	0.31	0.34	10.66	6.08	0.638
K5.1	0.83	−3.68	0.47	43.14	** < 0.001**
K5.2	0.44	0.08	1.93	39.16	** < 0.001**
K5.3	0.31	0.57	1.73	41.63	** < 0.001**
K5.4	0.34	0.48	1.60	44.05	** < 0.001**
K6.1	0.84	−4.67	0.38	11.57	0.172
K6.2	0.96	−6.95	0.47	22.44	**0.004**
K6.3	0.44	1.29	0.17	25.46	**0.001**
K6.4	0.99	−5.16	0.93	5.25	0.731
K6.5	0.94	−4.28	0.70	24.84	**0.002**
K6.6	0.75	−3.11	0.38	14.60	0.067
K6.7	0.62	−0.63	1.30	40.37	** < 0.001**
K6.8	0.74	32.21	-0.03	18.06	**0.021**
K7	0.19	3.29	0.44	4.47	0.813
K8.1	0.68	−1.72	0.52	18.09	**0.021**
K8.2	0.87	−3.37	0.65	13.51	0.095
K8.3	0.44	0.85	0.26	9.13	0.332
K8.4	0.32	0.82	0.93	10.59	0.226
K10	0.66	−3.96	0.17	17.33	**0.027**
K11	0.90	−5.50	0.41	10.56	0.228

Bold values indicate *p* < 0.05; statistically significant

**Table 4:** Unidimensionality test for all 2-PL IRT models

Model	Items	*P*
1	All items in the knowledge section	0.01
2	Excluding 2 items that are too difficult and with low discrimination and poor item fit: K1.9 and K6.8	0.01
3	Excluding 7 items that are too easy or too difficult and with poor item fit: K1.4, K1.9, K5.1, K6.2, K6.5, K6.8, K10	0.01
4	Excluding 9 items with low discrimination and with poor item fit:nK1.9, K1.10, K1.11, K1.15, K1.16, K1.17, K6.3, K6.8, K10	0.01
5	Excluding all 25 items with poor item fit: K1.4, K1.5, K1.6, K1.7, K1.8, K1.9, K1.10, K1.11, K1.14, K1.15, K1.16, K1.17, K3.1, K3.2, K5.1, K5.2, K5.3, K5.4, K6.2, K6.3, K6.5, K6.7, K6.8, K8.1, K10	0.63
6	Including 19 items with good item fit + 6 items with poor item fit but with acceptable difficulty and discrimination and considered important: K1.1, K1.2, K1.3, K1.12, K1.13, K2, K3.3, K4.1, K4.2, K4.3, K4.4, K6.1, K6.4, K6.6, K7, K8.2, K8.3, K8.4, K11 + K5.1, K5.2, K5.3, K5.4, K6.7, K8.1	0.29

**Table 5:** IRT analysis of final 25 items retained in the knowledge section

Item	Difficulty index	Discrimination index	Chi-square value	*P*
K1				
K1.1	−5.24	0.66	10.34	0.242
K1.2	−7.58	0.24	15.34	0.053
K1.3	−3.15	0.36	10.59	0.226
K1.12	1.31	0.61	10.36	0.241
K1.13	1.81	0.73	5.99	0.648
K2	5.52	0.02	2.76	0.948
K3				
K3.3	−7.33	0.54	6.36	0.607
K4				
K4.1	0.36	7.69	9.34	0.314
K4.2	−5.86	0.44	13.23	0.104
K4.3	0.37	9.37	9.19	0.327
K4.4	0.34	10.66	6.08	0.638
K5				
K5.1	−3.68	0.47	43.14	** < 0.001**
K5.2	0.08	1.93	39.16	** < 0.001**
K5.3	0.57	1.73	41.63	** < 0.001**
K5.4	0.48	1.60	44.05	** < 0.001**
K6				
K6.1	−4.67	0.38	11.57	0.172
K6.4	−5.16	0.93	5.25	0.731
K6.6	−3.11	0.38	14.60	0.067
K6.7	−0.63	1.30	40.37	** < 0.001**
K7	3.29	0.44	4.47	0.813
K8				
K8.1	−1.72	0.52	18.09	**0.021**
K8.2	−3.37	0.65	13.51	0.095
K8.3	0.85	0.26	9.13	0.332
K8.4	0.82	0.93	10.59	0.226
K11	−5.50	0.41	10.56	0.228

Bold values indicate *p* < 0.05; statistically significant

### Attitude

The KMO value for the attitude section was > 0.7, indicating that data are suitable for factor analysis. Based on communalities, there were six items (A1, A3, A5, A6, A9 and A13) that had very low h2 values and thus were excluded from further analysis (as they would not correlate with other items that explain attitudes regarding dengue). Two items (A4 and A14) had a low factor loading (< 0.4) but they were retained in the analysis as a factor loading > 0.3 is considered as the minimal accepted value. Three items (A4, A8 and A14) had a low correlation (< 0.5) with the total, as shown in the corrected item-total correlations. Interestingly, by excluding item A4 the overall Cronbach’s alpha score increased while it remained the same after excluding items A8 and A14. Due to this, only item A4 was excluded from the analysis. Overall, only eight items (A2, A7, A8, A10, A11, A12, A14 and A15) retained in the attitude section were considered as reliable (Table 6).

**Table 6:** The final eight items retained in the attitude section

Attitude towards dengue prevention	EFA	
Item	Factor loading	Reliability (Cronbach’s alpha score)
A2	0.68	0.83
A7	0.80	
A8	0.41	
A10	0.86	
A11	0.51	
A12	0.61	
A14	0.37	
A15	0.74	

### Practice

For the practice section, the data were suitable for factor analysis as the KMO value was > 0.7. Seven items (P9, P10, P11, P12, P14, P16 and P18) had low h2 values (< 0.3) and thus were excluded from further analysis. All remaining items had acceptable factor loading values (> 0.4) and corrected item-total correlation values (> 0.5). In conclusion, 11 items (P1, P2, P3, P4, P5, P6, P7, P8, P13, P15 and P17) were retained in the practice section (Table 7).

**Table 7:** The final 11 items retained in the practice section

Practice to prevent or reduce *Aedes* mosquito adults and larvae	EFA	
Item	Factor loading	Reliability (Cronbach’s alpha score)
P1	0.67	0.86
P2	0.77	
P3	0.76	
P4	0.63	
P5	0.69	
P6	0.55	
P7	0.52	
P8	0.56	
P13	0.56	
P15	0.50	
P17	0.50	

### Treatment-seeking behaviour

In comparison to the attitude and practice sections, the KMO value of treatment-seeking behaviour section was < 0.7. Hence, this section was not suitable for factor analysis as the items did not measure an underlying construct that can explain behaviour regards dengue. However, factor analysis was still conducted to confirm the results. The factor loading and corrected item-total correlation values for four items (B3, B4, B5 and B6) were not acceptable as their factor loadings of > 0.4 and corrected item-total correlation of > 0.5, which was considered not reliable. Hence, this section was removed from further analysis.

## Discussion

To the best of our knowledge this is the first report to describe the development and assessment of content validity, face validity, constructs validity and reliability of a KAP questionnaire for the resident population of Sabah, Malaysia, in order to inform the planning of an effective local health intervention for dengue infection. Overall, the results of the IRT analysis for knowledge and the EFA for attitude and practice indicated that the measurement model for each construct should undergo modification to improve the model fit. The final version of the KAP questionnaire consisted of 25 knowledge items, eight attitude items and 11 practice items, each of which is reliable and valid for the Sabah population.

The IRT analysis of the knowledge section found several items to be problematic in terms of their relevance to measuring knowledge of dengue infection. As such, those items were excluded from further analysis. The ideal parameter range for discrimination values is from minus infinity to plus infinity; however, questions with negative figures of discrimination are recognized as problematic because they infer that participants with a high score are less expected to support more stringent response alternatives [28]. The items retained in the final model were based on unidimensionality, where the P-value is more than α (> 0.05). All 25 retained items (out of 44) in the knowledge section had acceptable difficulty and discrimination values. Moreover, the average Cronbach’s alpha score was 0.75, indicating that the knowledge section of the questionnaire may be considered as reliable [29]. This value is comparable to those obtained from previously published validation studies on KAP in Malaysia [30, 31].

For the attitude section, eight out of 15 items were retained, revealing close relations between factors and items [32]. Six items were excluded from further analysis as very low h2 values indicated that they are not correlated with other items that explain attitudes towards dengue prevention. In addition, one item was excluded in spite of having the minimally accepted factor loading value because the overall Cronbach’s alpha score increased when it was removed from analysis. The reliability analysis of items retained in the attitude section indicated an acceptable Cronbach’s alpha value of 0.83, demonstrating internal consistency. This internal consistency for attitude was higher than that obtained in a prior study performed in Perak state, Malaysia (Cronbach's alpha = 0.638) [33].

In regard to practice, 11 out of 18 items were retained as each having an acceptable factor loading value (> 0.4) and corrected item-total correlation value (> 0.5). In some KAP survey guidelines it is recommended that developers start with a large number of items and apply item reduction techniques to select a small number of final items [13]. The 11 items retained in the practice section were reliable with acceptable a Cronbach’s alpha score of 0.86 demonstrating internal consistency. This value is higher than that attained in the one previous study reported in Malaysia, undertaken in Perak state (0.79) [33].

Lastly, the treatment-seeking behaviour section showed poor psychometric properties. The factor analysis was not suitable, while the reliability analysis indicated an unacceptable Cronbach’s alpha score, demonstrating low internal consistency. Hence, this section was removed from analysis.

The present study has several limitations. Firstly, the participants were recruited only from Sabah, Malaysia, as the questionnaire was intended to be used specifically for the Sabahan population. Thus, cross-validation studies are needed in other parts of East Malaysia, as well as extended to Peninsular Malaysia, for application of the questionnaire among other communities. Secondly, IRT and EFA were used in this study to assess reliability and validity; however, it is recommended that in future confirmatory factor analysis should be conducted to validate the EFA model. Thirdly, the initial design of the questionnaire allowed respondents to choose a “don’t know” response for any item. In a number of cases, this action may have excluded them from being taking into account when calculating a total score, which in turn prevented a better determination of the KAP. In retrospect, a “best guess” response option is more helpful than a non-answer to diagnosis, and therefore we have made this minor modification to the questionnaire.

Although this is a convenience sampling, in order to ensure that the data collection was representative respondents were recruited from across the entire state. Ethnicity is the foremost sample characteristic that may not be representative. Sabah is multiethnic, with ethnicity distributions that are not equal among the state's five divisions. For example, Rungus ethnics are more prevalent in Kudat division, Kadazandusun, Brunei, Bajau, and Sabah Malay in West Coast division, while Bugis and Suluk ethnics are located predominantly in Tawau & Sandakan division. In addition, there are smaller minority groups in Sabah that are defined simply as “others” in Malaysian census data. Hence, it cannot be excluded that the study's ethnicity distribution is not representative of the Sabah population as a whole. Such non-representativeness may have biased the outcomes. We acknowledge the particular importance of representativeness when estimating item discrimination and difficulty as both may depend on the specific sample.

## Conclusion

In this study a new KAP questionnaire on dengue was developed and validated for the Sabah population in Malaysia, which may serve as a survey reference for researchers wishing to study Sabahan communities. In our validated KAP questionnaire, the final model has four sections and comprises 50 items, consisting of six items on sociodemographic information, 25 items on knowledge, eight items on attitude and 11 items on preventive practice. Evidence from IRT analysis and EFA indicates that the knowledge, attitude and practice sections are psychometrically valid, while each also demonstrates good reliability. However, the psychometric properties of the treatment-seeking behaviour section are unsatisfactory, suggesting that further development of this section is warranted in future studies. Moreover, further investigation, such as confirmatory factor analysis, is warranted to confirm the KAP scales.

## Supplementary Information

**Additional file 1.** Questionnaire use to assess knowledge, attitude, preventive practice, and treatment seeking behaviour on dengue among residents of Sabah population.

## Data Availability

All data generated or analysed during this study are included in this published article [and its Additional file 1].

## Abbreviations

KAP: Knowledge, attitude and preventive practice
2-PL IRT: Two-parameter logistic item response theory
KMO: Kaiser-Meyer-Olkin
EFA: Exploratory factor analysis
