# Supplementary material for: Development and validation of a structured survey questionnaire on knowledge, attitude, preventive practice, and treatment-seeking behaviour regarding dengue among the resident population of Sabah, Malaysia: an exploratory factor analysis
Source: BMC Infect Dis. 2021 Aug 31;21:893. doi: 10.1186/s12879-021-06606-6 (PMC8406825; doi:10.1186/s12879-021-06606-6)
Supplement: Supplementary file 1 — Additional file 1. Questionnaire use to assess knowledge, attitude, preventive practice, and treatment seeking behaviour on dengue among residents of Sabah population. [file 12879_2021_6606_MOESM1_ESM.docx]

*Good day,*

*We are researchers from the Faculty of Medicine & Health Sciences Universiti Malaysia Sabah (UMS) conducting a study on* ***Knowledge, Attitude and Practice towards Dengue in Sabah.***

The survey study was approved by the UMS ethics committee [Ethics reference number: (53) dlm.JKN (SB)100-13] and complies with the Declaration of Helsinki. Permission to interview respondents was obtained from the district police station [KPB Kota Kinabalu 10/8/11].

**Investigators:**

**Rhanye Mac Guad:** Department of Biomedical Science and Therapeutics, Faculty of Medicine and Health Sciences, Universiti Malaysia Sabah, Kota Kinabalu 88400, Malaysia

**Mohammad Saffree Jeffree:** Department of Community and Family Medicine, Faculty of Medicine and Health Science, Universiti Malaysia Sabah, Kota Kinabalu 88400, Malaysia

**Nelbon Giloi:** Department of Community and Family Medicine, Faculty of Medicine and Health Science, Universiti Malaysia Sabah, Kota Kinabalu 88400, Malaysia

**Chandrika Murugaiah:** Department of Biomedical Science and Therapeutics, Faculty of Medicine and Health Sciences, Universiti Malaysia Sabah, Kota Kinabalu 88400, Malaysia

**Nornazirah Azizan:** Department of Pathobiology and Medical Diagnostic, Faculty of Medicine and Health Science, Universiti Malaysia Sabah, Kota Kinabalu 88400, Malaysia

**RESPONDENT INFORMATION**

Please answer all questions below

1. Age: ……...(years)
2. Gender: Male/Female………………………………………………………………………….....
3. Address:…………………………………………………………………………………………..
4. Ethnicity: Kadazan-Dusun, Bajau, Brunei, other – please specify………………………………

1. Marital status: Single, Married, other – please specify…………………………………………...
2. Highest education level: No education, Primary, Secondary, College/University..........................
3. Occupation: Unemployed, Self-employed, Employed – please state type of education …………………………………………………………………………………………………….

**PART A: GENERAL KNOWLEDGE OF DENGUE**

**Please answer all questions below by tick (✓).**

1. **Which one or more of the following is a sign of dengue?**

| **No.** | **Sign** | **Yes** | **No** | **Don’t know** |
| --- | --- | --- | --- | --- |
| 1 | Fever |  |  |  |
| 2 | Chills |  |  |  |
| 3 | Nausea and vomiting |  |  |  |
| 4 | Headache |  |  |  |
| 5 | Joint pain |  |  |  |
| 6 | Muscle pain |  |  |  |
| 7 | Pain behind eyes |  |  |  |
| 8 | Painful backbone |  |  |  |
| 9 | Stomach pain |  |  |  |
| 10 | Bleeding of nose, gums and skin |  |  |  |
| 11 | Skin rash |  |  |  |
| 12 | Cough |  |  |  |
| 13 | Swollen glands |  |  |  |
| 14 | Restlessness |  |  |  |
| 15 | Pale or cold to touch |  |  |  |
| 16 | Respiratory disturbances |  |  |  |
| 17 | Circulatory disturbances |  |  |  |

**2. Is dengue a contagious disease?**

| **Yes** | **No** | **Don’t know** |
| --- | --- | --- |
|  |  |  |

**3. Which one or more of the following insects is the carrier of dengue?**

| **No.** | **Carrier** | **Yes** | **No** | **Don’t know** |
| --- | --- | --- | --- | --- |
| 1 | Flies |  |  |  |
| 2 | Ticks |  |  |  |
| 3 | Mosquitoes |  |  |  |

**4. What one or more type of mosquito carries dengue?**

| **No.** | **Type of mosquito** | **Yes** | **No** | **Don’t know** |
| --- | --- | --- | --- | --- |
| 1 | *Culex* |  |  |  |
| 2 | *Aedes* |  |  |  |
| 3 | *Anopheles* |  |  |  |
| 4 | *Mansonia* |  |  |  |

**5. At what time of day are dengue carriers more likely to cause infection in humans?**

| **No.** | **Blood sucking time** | **Yes** | **No** | **Don’t know** |
| --- | --- | --- | --- | --- |
| 1 | Dawn and late afternoon |  |  |  |
| 2 | Morning and noon |  |  |  |
| 3 | Evening and midnight |  |  |  |
| 4 | All the time |  |  |  |

**6. Which one or more of the following is a breeding place for dengue carriers?**

| **No.** | **Breeding place** | **Yes** | **No** | **Don’t know** |
| --- | --- | --- | --- | --- |
| 1 | Discarded food container |  |  |  |
| 2 | Discarded tyre |  |  |  |
| 3 | Tree branch |  |  |  |
| 4 | Unclosed water reservoir |  |  |  |
| 5 | Flowerpot |  |  |  |
| 6 | Open pool of water |  |  |  |
| 7 | Flowing water |  |  |  |
| 8 | Puddle |  |  |  |

**7. Are there specific treatments for dengue?**

| **Yes** | **No** | **Don’t know** |
| --- | --- | --- |
|  |  |  |

**8. Which one of more of the following is a treatment for dengue?**

| **No.** | **Treatment** | **Yes** | **No** | **Don’t know** |
| --- | --- | --- | --- | --- |
| 1 | Plenty of rest |  |  |  |
| 2 | Drinking water abundantly |  |  |  |
| 3 | Taking panadol (paracetamol) |  |  |  |
| 4 | Traditional herbal remedies |  |  |  |

**9. How do you receive information on dengue?**

| **No** | **Source of information** | **Yes** | **No** |
| --- | --- | --- | --- |
| 1 | Books/newspapers/pamphlets |  |  |
| 2 | Media mass (TV, Radio) |  |  |
| 3 | Internet |  |  |
| 4 | Health profession staff |  |  |
| 5 | Public health campaign |  |  |
| 6 | People in the local community |  |  |

**10. Is the rainy season when dengue cases most frequently occur?**

| **Yes** | **No** | **Don’t know** |
| --- | --- | --- |
|  |  |  |

**11. Can fines be imposed if mosquito larvae are found on your residential property?**

| **Yes** | **No** | **Don’t know** |
| --- | --- | --- |
|  |  |  |

**PART B: ATTITUDE TOWARDS DENGUE PREVENTION**

**Please answer all questions below by tick (✓).**

| **No.** | **Question** | **Strongly agree** | **Agree** | **Not sure** | **Not agree** | **Strongly not agree** |
| --- | --- | --- | --- | --- | --- | --- |
| 1 | Dengue is a serious illness |  |  |  |  |  |
| 2 | Dengue is a type of disease that cannot be prevented |  |  |  |  |  |
| 3 | Everyone is at risk from dengue |  |  |  |  |  |
| 4 | Children are especially susceptible to dengue |  |  |  |  |  |
| 5 | Dengue can be repeated (multiple infections) |  |  |  |  |  |
| 6 | Early stages of dengue are fully treatable |  |  |  |  |  |
| 7 | Disposal of mosquito larvae is a waste of time and troublesome |  |  |  |  |  |
| 8 | Killing mosquitoes carrying dengue is the only way to control or prevent infection |  |  |  |  |  |
| 9 | I can play an important part to prevent dengue |  |  |  |  |  |
| 10 | Eradicating mosquito breeding grounds is the responsibility of health professionals and volunteers only |  |  |  |  |  |
| 11 | Supervised control and monitoring of suspected mosquito breeding grounds should be undertaken at least once a year |  |  |  |  |  |
| 12 | 'Fogging' can prevent mosquito breeding completely |  |  |  |  |  |
| 13 | If you had any signs of dengue would you see a doctor? |  |  |  |  |  |
| 14 | Fines can help towards control of dengue |  |  |  |  |  |
| 15 | Health professionals are not required to inspect residential properties |  |  |  |  |  |

**PART C: PRACTICE TO PREVENT OR REDUCE *AEDES* MOSQUITO ADULTS AND LARVAE**

**Please answer all questions below by tick (**✓**).**

| **No.** | **Question** | **Yes** | **No** | **Not sure** |
| --- | --- | --- | --- | --- |
| 1 | Are containers of water/wells in your residence covered? |  |  |  |
| 2 | Do you close water containers/wells once you have finished using them? |  |  |  |
| 3 | Do you always check the condition of stored water in containers/wells if you do not use them for more than five days? |  |  |  |
| 4 | Do you put an abate in the reservoir in your residence? |  |  |  |
| 5 | Have you ever checked for mosquito larvae in a discarded flower vase? |  |  |  |
| 6 | If there is stagnant water in a flower vase, would you throw it away? |  |  |  |
| 7 | Have you ever checked your residence and property for a container/place that could provide a mosquito breeding site? |  |  |  |
| 8 | Do you remove containers that can collect water and allow it to stagnate? |  |  |  |
| 9 | Do all members of your household sleep under a mosquito net each night? |  |  |  |
| 10 | Does your residence have window nets/screens? |  |  |  |
| 11 | Do all members of your family/household use mosquito coils each night? |  |  |  |
| 12 | Do all members of your family/household use mosquito sprays each night? |  |  |  |
| 13 | Do you check and clean the gutters of your residence each rainy season? |  |  |  |
| 14 | Do your family/household members take part in campaigns to prevent *Aedes* mosquito breeding in your local community? |  |  |  |
| 15 | Do you consider eradicating *Aedes* mosquitoes is a shared responsibility? |  |  |  |
| 16 | If there is a gotong-royong in your local community, would you invite all family/household members to join? |  |  |  |
| 17 | Would you allow the authorities to conduct dengue prevention activities at your residence? |  |  |  |
| 18 | Do you and your family/household members always wear long-sleeved clothing if forced out of the house at dusk. |  |  |  |

**PART D: TREATMENT-SEEKING BEHAVIOR RELATED TO DENGUE**

Instructions: Please tick the column which best corresponds your answer for the scenario below:

***You suddenly become restless and lethargic. What would you do?***

| No. | **Statement** | **Yes** | **No** |
| --- | --- | --- | --- |
| 1 | Take yourself immediately to the nearest hospital |  |  |
| 2 | Take yourself to a general practitioner near to your residence |  |  |
| 3 | Phone an ambulance |  |  |
| 4 | Wait for a few hours to see if symptoms improve |  |  |
| 5 | Take antipyretics e.g. panadol and wait to see if symptoms improve |  |  |
| 6 | Take natural treatments/traditional herbal remedies for dengue immediately |  |  |

***Thank you for your cooperation***

***~Working together to eradicate dengue~***
